# Anxiety, depression, and quality of life in children and adults with alopecia areata: A systematic review and meta-analysis

**DOI:** 10.3389/fmed.2022.1054898

**Published:** 2022-11-29

**Authors:** Marije van Dalen, Kirsten S. Muller, Johanna M. Kasperkovitz-Oosterloo, Jolanda M. E. Okkerse, Suzanne G. M. A. Pasmans

**Affiliations:** ^1^Department of Pediatric Gastroenterology, Erasmus MC Sophia Children’s Hospital, Rotterdam, Netherlands; ^2^Department of Child and Adolescent Psychiatry/Psychology, Erasmus MC Sophia Children’s Hospital, Rotterdam, Netherlands; ^3^Dutch Alopecia Association, ’s-Hertogenbosch, Netherlands; ^4^IPSO Institutes for Psychosocial Oncology, Almere, Netherlands; ^5^Department of Dermatology, Center of Pediatric Dermatology, Erasmus MC Sophia Children’s Hospital, Rotterdam, Netherlands

**Keywords:** alopecia, alopecia areata, psychosocial functioning, anxiety, depression, quality of life, meta-analysis

## Abstract

**Introduction:**

Alopecia areata (AA) is a non-scarring hair loss condition, subclassified into AA, alopecia universalis, and alopecia totalis. There are indications that people with AA experience adverse psychosocial outcomes, but previous studies have not included a thorough meta-analysis and did not compare people with AA to people with other dermatological diagnoses. Therefore, the aim of this systematic review and meta-analysis was to update and expand previous systematic reviews, as well as describing and quantifying levels of anxiety, depression, and quality of life (QoL) in children and adults with AA.

**Methods:**

A search was conducted, yielding 1,249 unique records of which 93 were included.

**Results:**

Review results showed that people with AA have higher chances of being diagnosed with anxiety and/or depression and experience impaired QoL. Their psychosocial outcomes are often similar to other people with a dermatological condition. Meta-analytic results showed significantly more symptoms of anxiety and depression in adults with AA compared to healthy controls. Results also showed a moderate impact on QoL. These results further highlight that AA, despite causing little physical impairments, can have a significant amount on patients’ well-being.

**Discussion:**

Future studies should examine the influence of disease severity, disease duration, remission and relapse, and medication use to shed light on at-risk groups in need of referral to psychological care.

**Systematic review registration:**

[https://www.crd.york.ac.uk/prospero/], identifier [CRD42022323174].

## Introduction

Alopecia areata (AA) is a hair loss condition with a lifetime prevalence of 2.1% (1). AA has a peak onset between 25 and 29 years old, with a median age at diagnosis of 31 for males and 34 for females. It occurs more frequently in people with a non-white ethnicity (2). Males and females appear to be affected equally often (2), however research has also reported females to be slightly more likely to experience AA (2). AA is typically divided into AA (patchy hair loss), alopecia universalis (AU; total loss of scalp hair), alopecia totalis (AT; total loss of body hair) and alopecia ophiasis (band-like hair loss on the temporal and occipital scalp) (3).

Alopecia areata has an unpredictable disease course characterized by relapse and remission (4). Full hair regrowth may be observed in 50–80% of patients (5, 6), but relapse rates of 30–52% have been reported (5) with around 30% of patients with AA eventually progressing to complete hair loss (6). Relapse is more likely in patients with an earlier onset of AA, but is not related to gender, clinical severity and treatment given (5). Furthermore, medication often fails to provide sustained hair regrowth (3).

There are indications that people with AA experience adverse psychosocial outcomes. Qualitative studies, for instance, have shown that patients reported considerable distress (7). Feelings of sadness, insecurity, inadequacy, and self-consciousness (8), as well as feelings of depression, anxiety, and suicidal thoughts (7) were prevalent. The majority of qualitative research highlights that people struggle with everyday activities, such as participating in sports or social events, due to a fear of their appearance being noticed (7–9). The unpredictable nature of AA was also highlighted as a source of distress in particular (7, 8) and women seem to report more stress and distress than men (10, 11).

Most quantitative research has focused on anxiety, depression or quality of life (QoL). For anxiety, a meta-analysis including eight studies by Okhovat et al. (12) showed that people with AA are 2.50 times more likely to experience anxiety. However, it is unclear how the papers were selected and what type of control group was included in the meta-analysis. Other studies have shown that people with AA have a higher chance of being diagnosed with an anxiety disorder than healthy controls (13). When the amount of anxiety symptoms of people with AA is compared to people with other dermatological diagnoses mixed results have been found (14).

When looking at depression, the aforementioned meta-analysis found that people with AA are 2.71 times more likely to experience depression (12). This result is corroborated by other studies reporting people with AA to be more likely to be diagnosed with depression (13, 15). As for anxiety, it is unclear how people with AA compare to people with other dermatological diagnoses (16, 17).

A systematic review conducted in 2018 has shown that AA has a considerable impact on QoL (18). However, it remained unclear how QoL was related to disease severity (18). Furthermore, people with AA were not compared to people with different dermatological diagnoses in this review. More recent research has reported a moderate effect on QoL (19), as well as no effect (20). Comparisons to people with a different dermatological diagnosis have yielded mixed results. For instance, one study comparing people with AA to people with alopecia androgenetica reported people with AA to have better QoL (21), while another study found the opposite result (22).

Although previous systematic reviews on psychosocial consequences of AA have been conducted (e.g., 18, 23), it remains unclear how people with AA compare to people without AA or people with a different dermatological diagnosis. In addition, these reviews have not highlighted the psychosocial impact of AA on different age groups (i.e., children or adults). Therefore, the purpose of the current systematic review and meta-analysis was to update and expand previous systematic reviews, as well as describing and quantifying levels of anxiety, depression, and QoL in patients with AA, AU, or AT. We also aimed to explore whether gender or age would influence the amount of anxiety, depression, and QoL experienced by people with AA. We specifically sought to answer the following research question: What is the impact of living with alopecia areata, alopecia totalis or alopecia universalis on levels of anxiety, depression, and quality of life in children and adults? We also wanted to know how levels of anxiety, depression, and QoL of people with AA compared to people with a different dermatological condition and to healthy controls.

## Materials and methods

**FIGURE 1:**
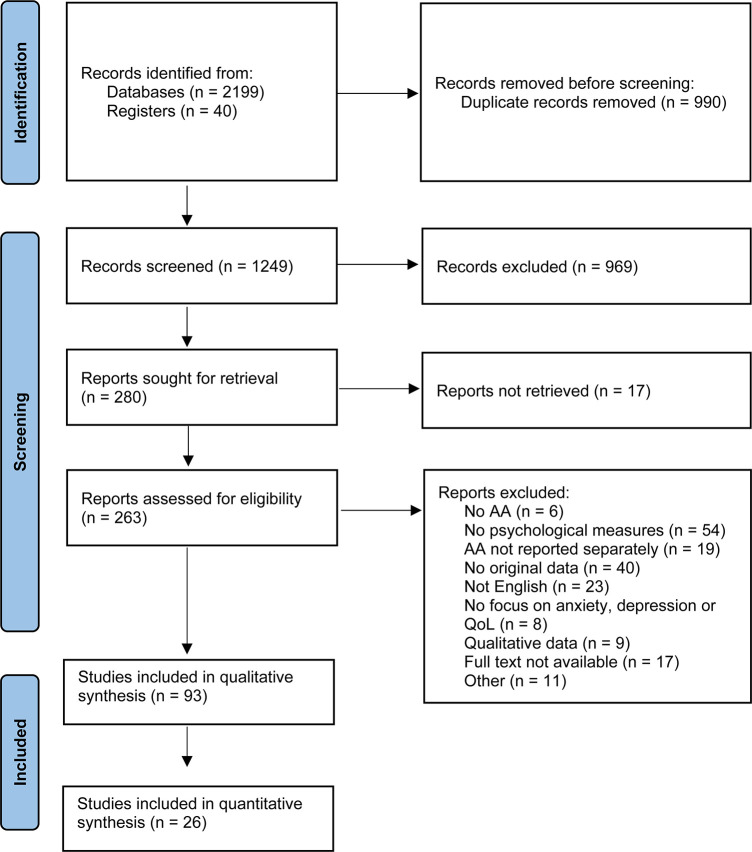
PRISMA flowchart of the selection process.

This article was written in accordance with the Preferred Reporting Items for Systematic Reviews and Meta-Analyses (PRISMA) statement (24) and was registered prospectively in the international prospective register of systematic reviews, PROSPERO, registration number CRD42022323174. The protocol was registered with a broad focus on psychological impact of AA, as it was unclear how many papers the search would yield. After selection of relevant papers, a decision was made to focus only on anxiety, depression and QoL and a further nine papers were excluded (see Figure 1).

### Search strategy

As this article was part of a bigger project for the Dutch Alopecia Association, a broad search focusing on the psychosocial impact of living with AA was conducted by a research librarian on 28 March 2022. The following databases were searched from inception: Embase, Medline, Web of Science Core Collection, Cochrane Central Register of Controlled Trials, PsycInfo, and Google Scholar. The search included terms, both Mesh and free text, related to alopecia and the psychosocial impact, without restrictions on language or publication date. Only published, peer-reviewed papers were used. The full search is displayed in Supplementary material.

### Eligibility criteria

Studies were included if they met the following eligibility criteria: (a) studied a sample with AA, AU, and/or AT, (b) reported quantitative data on anxiety, depression or QoL, and (c) the paper was an original research paper. Studies were excluded if they (a) reported no original data (e.g., case-reports, conference abstracts, and systematic reviews), (b) were not written in English, or (c) did not separate AA from other medical diagnoses. No criteria were set for the amount of timepoints in an article (i.e., the article being cross-sectional or longitudinal). In case of a longitudinal intervention study, only the baseline data were included.

### Study selection

Studies were selected if they met the inclusion and exclusion criteria. Two reviewers (MD and KM) independently assessed the title and abstract. The interrater agreement was 81.55%. Discrepancies were resolved using consensus. Afterward, the two reviewers independently assessed the full text for eligibility. Interrater agreement for this step was 87.46%. Discrepancies were again resolved using consensus. One of the reviewers (MD) checked the reference list of included articles for additional relevant references. Any references deemed relevant were first screened based on title and abstract. If still relevant, the full-text was read. When the article met the inclusion and exclusion criteria, it was included in the review. Endnote 20 was used to manage references.

### Data extraction

Data collection was done by one researcher (MD) and checked by another researcher (KM) using a data extraction form. The following data were extracted: type of alopecia, sample size, percentage male, mean age (SD), age range, method involved (questionnaire or interview), main conclusions, mean score (when method is questionnaire), mean prevalence of symptoms/diagnosis, any relevant comparisons between groups (e.g., anxiety symptoms in AA vs. unaffected controls). Authors of papers were contacted when relevant data for meta-analyses was missing.

### Quality and risk of bias

Quality and risk of bias were assessed using the relevant NIH quality assessment tool for controlled intervention studies, observational cohort and cross-sectional studies, case-control studies or before-after studies with no control group [National Heart, Lung, and Blood Institute (NIH), 2018] (25) or the QAVALS (26). Questions can be answered with “yes, no or cannot determine/not reported/not applicable responses.” We rated >80% points as good, 60–80% points as fair and <60% as poor quality. Quality assessment was performed independently by two reviewers (MD and KM). Half of the articles were discussed in a consensus meeting, after which the remaining half of the papers was checked by one reviewer (MD).

### Data synthesis and statistical analyses

All studies were included in the qualitative synthesis. Meta-analyses were conducted for five or more similar studies. As a high level of between-study heterogeneity was expected, a random-effects model was used to pool effect sizes. The Restricted Maximum Likelihood Estimator (REML) was used to calculate heterogeneity variance (27). Means and standard deviations (SDs) of samples were used to compute effect sizes, the standardized mean differences (SMDs), quantified in the form of Hedges’ *g* (28). When means and SDs were not available, medians were transformed to means and SDs as described by Shi et al. (29). Publication bias was tested by visual inspection of a contour-enhanced funnel plot (30) and Egger’s test in case of ≥10 studies. Exploratory meta-regressions were conducted. For each meta-analysis, one model was created with mean age, percentage male, and quality rating as independent variables. The significance level was set to α = 0.05. Analyses were done using the meta package (31) in RStudio.

## Results

### Study selection

After removing duplicate records, a total of 1,249 records were retrieved for screening. After title and abstract screening, 280 records were assessed for eligibility. Finally, 93 articles were included for the qualitative synthesis of which 26 articles were also included in the quantitative synthesis. The full selection process is displayed in Figure 1. Overall, 74 papers were of poor quality, 16 papers were of fair quality and 4 papers were of good quality.

A total of 52 papers studied anxiety in children and/or adults with AA. Seven papers (32–38) had a combined research group with children and adults (*n* = 11,007, *M*_age_ = 41.78, 43.63% male), eight papers (39–46) studied children with AA (*n* = 398, *M*_age_ = 11.85, 47.00% male) and 37 papers (13, 14, 17, 19, 22, 37, 38, 47–77) studied adults with AA (*n* = 88,858, *M*_age_ = 40.03, 41.25% male).

For depression, 65 papers were included. Fourteen papers (32–36, 38, 78–84) looked at children and adults (*n* = 18.638, mean age = 36.26, 43.44% male), nine papers (39–46, 85) studied children (*n* = 3908, *M*_age_ = 11.85, 44.82% male) and 42 papers (13–17, 19, 22, 47–53, 55–66, 68–77, 86–90) studied adults with AA (*n* = 93,047, *M*_age_ = 41.69, 40.39% male).

A total of 40 studies investigated QoL in people with AA. Five studies (36, 78, 83, 91, 92), combined children and adults into one sample (*n* = 3611, *M*_age_ = 31.43, 60.09% male), three studies (41, 43, 93) investigated children (*n* = 258, *M*_age_ = 11.50, 47.45% male) and 32 studies (17, 19–22, 53, 61, 62, 67, 73, 75, 76, 94–113) investigated adults with AA (*n* = 5,373, *M*_age_ = 41.38, 42.83% male).

### Anxiety

The results for anxiety are shown in Table 1.

**TABLE 1:** Results for anxiety.

References	Country	Year	*N*	% male	Age (*M*, SD)	% AA, AT, AU	Controls	Measures	Conclusions	Quality score (%)
**Pediatric and adult samples**										
Ataseven et al. (32)	Turkey	NR	43	72.1	23.42 (11.41)	NR	30 healthy controls	HAM-A	AA more symptoms of anxiety than healthy controls	30^3^
Chu et al. (37)	Taiwan	2000–2009	5,117	49.2	NR	NR	20,468 healthy controls	ICD-9 codes	AA diagnosed with anxiety more often than controls	80^3^
Kökcam et al. (33)	Turkey	NR	17	NR	26.47 (12.2)	NR	11 vitiligo, 20 healthy controls	SCL-90-R	AA more symptoms of anxiety than healthy controls, no differences with vitiligo	20^3^
Marahatta et al. (38)	Nepal	August 2015–July 2016	75	53.3	29.40 (9.90)	NR	No	BAI	89.0% very low anxiety, 8.0% moderate anxiety, 0% severe anxiety	45.83^4^
Singam et al. (34)	USA	2002–2012	5,605 hospitalized patients	38.3	42.2 (NR)	NR	Hospitalized patients without AA (N unknown)	ICD-9 codes	AA diagnosed with anxiety more often than controls	45^3^
Talaei et al. (35)	Iran	April–July 2005	24	33.33	25.38 (8.32)	NR	24 healthy controls	SCL-90-R	No significant difference with controls	70^3^
Vélez-Muñiz et al. (36)	Mexico	March 2017–February 2018	32 child, 94 adults	41	NR	92.9% patchy AA, 3.2% AT, 1.6% ophiasis, 1.6% AU	No	HADS	For adults: 19.1% heightened anxiety/depression, 34.1% no anxiety/depression	50^4^
**Pediatric samples**										
Altunisik et al. (39)	Turkey	NR	27	29.6	11.9 (3.3)	85.19% AA, 14.81% AU	30 dermatology patients	K-SADS-PL; SCARED; STAI-C	No difference with controls on questionnaires or diagnoses. 51.8% of AA patients had at least 1 anxiety diagnosis	65^3^
Andreoli et al. (40)	Italy	1997–2000	176	NR	NR	NR	No	Diagnosis by psychologist	16% diagnosis generalized anxiety disorder, 8% social anxiety disorder	25^4^
Bilgiç et al. (41)	Turkey	NR	74	55.41	12.1 (2.8)	NR	65 healthy controls	STAI-C	AA more state anxiety than controls. Children, but not adolescents more trait anxiety than controls.	65^3^
Díaz-Atienza and Gurpegui (42)	Spain	NR	31	52	12.2 (3.8)	51.61% AA, 48.39% AU/AT	23 epilepsy, 25 siblings	STAI-C	No difference on symptoms of anxiety between AA and epilepsy or sibling group	65^3^
Erdoğan and Gür (43)	Turkey	October 2018–December 2019	31	54.83	12.54 (3.56)	100% AA	29 vitiligo, 30 healthy controls	RCADS-C; RCADS-P	More social anxiety and total anxiety in AA (child-reported) for HC. More panic disorder and total anxiety in AA (parent-reported) for HC. No differences with vitiligo.	60^3^
Ghanizadeh (44)	Iran	August 2004–November 2006	14	NR	11.66 (6.08)	NR	No	K-SADS-PL	7.1% diagnosis social anxiety SAS, 28.6% specific phobia, 7.1% generalized anxiety disorder	50^4^
Liakopoulou et al. (45)	Greece	NR	33	30.3	10.5 (0.3)	NR	30 patients from pediatrician	CMAS	AA higher scores on worry, oversensitivity and concentration	40^3^
Reeve et al. (46)	USA	NR	12	NR	11.5 (2.9)	NR	No	DICA-R; RCMAS	58.33% with any anxiety disorder diagnosis	37.5^4^
**Adult samples**										
Aghaei et al. (47)	Iran	NR	40	44.8	35.2 (9.2)	NR	40 healthy controls	BAI	More symptoms of anxiety in AA patients than controls	35^3^
Alfani et al. (48)	Italy	November 2009–October 2010	73	45.2	35.2 (9.2)	61.7% AA, 26.0% AT, 12.3% AU	73 healthy controls	Clinical interview; MMPI-2	More anxiety in AA patients than controls	35^3^
Altinöz et al. (49)	Turkey	September 2011–October 2012	30	50	33.3 (8.9)	NR	30 urticaria, 39 healthy controls	HADS	More anxiety in AA patients than healthy controls. No difference with urticarial.	40^3^
Annagur et al. (50)	Turkey	NR	73	65.75	27.66 (7.79)	100% AA	78 healthy controls	SCL-90	No difference in symptoms of anxiety	35^3^
Atış et al. (19)	Turkey	NR	39	59	33.5 (11.6)	NR	46 vitiligo, 46 healthy controls	HADS	AA more anxiety than healthy controls. No difference with vitiligo.	20^3^
Baghestani et al. (51)	Iran	NR	68	72	35.4 (7.6)	100% AA	68 healthy controls	HAM-A	AA more symptoms of anxiety than healthy controls	60^3^
Bain et al. (52)	UK	NR	39	23.07	43.15 (12.43)	NR	23 PsA; 26 healthy controls	HADS^2^	More anxiety in less severe AA and shorter disease duration	30^3^
Balieva et al. (53)	13 European countries	November 2011–February 2013	33	33.3	42.8 (14.1)	NR	1,359 healthy controls	EQ-5D-3L	AA 4 times higher chance of anxiety/depression than controls	65^3^
Brajac et al. (54)	Croatia	1995–1999	45	37.78	40.24 (13.01)	100% AA	45 benign scalp lesions	STAI	AA more symptoms of anxiety than healthy controls	60^3^
Bukharia et al. (55)	India	NR	100	48	54% 15–30 years, 46% 31–50 years	NR	100 TE, 100 healthy controls	HAM-A	36.84% of AA and 43.94% of TE heightened anxiety	45^3^
Cakirca et al. (56)	Turkey	March–December 2017	33	75.8	26.33 (6.08)	NR	33 healthy controls	HADS	AA more symptoms of anxiety than healthy controls	30^3^
Colon et al. (57)	USA	April 1985–October 1987	31	29	35.70 (10.23)	74% AA, 23% AT, 42% AU^1^	No	DIS	Lifetime prevalence generalized anxiety disorder 39%, specific phobia 23%, panic disorder 13%	33.33^4^
Conic et al. (58)	USA	2005–2014	584	31.5	35.54 (19.28)	94.7% AA, 2.05% AT, 3.25% AU	172 SD	Diagnoses in patient file	No difference with SD. 13.70% of AA has any diagnosis of anxiety	35^3^
Cordan Yazici et al. (59)	Turkey	NR	43	60.5	33.80 (10.02)	95.35% AA, 4.65% AT	53 healthy controls	HADS	No significant differences between AA and controls	25^3^
Devar (60)	India	NR	30	100	NR	NR	30 TV, 30 healthy controls	TMAS	AA more symptoms of anxiety than healthy controls, no difference with TV	50^3^
Endo et al. (61)	Japan	June 2009–August 2010	122	33.1	38.3 (16.5)	NR	No	STAI	Anxiety not related to disease severity and disease duration	56.25^5^
Gallo et al. (77)	Italy	NR	16	37.5	45.95 (13.25)	NR	No	BSI	AA more symptoms of anxiety than norm group	39.29^6^
Güleç et al. (62)	Turkey	March 2001–January 2002	52	65.38	31.53 (12.61)	94.23% AA, 3.65% AU, 1.92% AT	52 healthy controls	BAI	No differences AA and controls	25^3^
Karia et al. (17)	India	NR	50	60.0	27.76 (NR)	NR	50 psoriasis, 50 healthy controls	DSM-IV-TR diagnosis	4% of AA any anxiety disorder diagnoses. More often than healthy controls, less often than psoriasis.	60^3^
Kim et al. (13)	South Korea	2002–2013	7,706	51.9	54.6% 20–39, 39.4% 40–59, 6.1% 60+	NR	30,824 without AA	ICD-10 codes	AA higher risk of anxiety disorder diagnosis than controls	65^3^
Kose et al. (63)	Turkey	NR	18	100	21.3 (NR)	NR	No	STAI	Positive correlation between anxiety and depression or hopelessness	50.00^7^
Macbeth et al. (64)	UK	January 2009–December 2018	5,435	45.9	38.93 (14.35)	NR	21,470 healthy controls	Diagnoses in patient file	3.24% of AA and 0.24% of healthy controls had anxiety disorder diagnoses	80^3^
Rajoo et al. (65)	Australia	NR	83	NR	40.95 (13.24)	NR	No	DASS-21	66.3% reported extreme symptoms of anxiety	54.17^4^
Ruiz-Doblado et al. (66)	Spain	NR	32	15	NR	NR	No	SCAN	22.2% diagnosis generalized anxiety disorder, 7.4% social phobia	37.5^4^
Russo et al. (67)	Italy	September 2016–September 2017	27	33.3	37.55 (10.37)	NR	80 AGA, 36 TE	STAI; SPS	No differences in trait anxiety or social anxiety. AA less social phobias than AGA and TE	50^3^
Şahiner et al. (68)	Turkey	August 2009–July 2010	41	49	32.9 (10.5)	NR	30 psoriasis, 50 healthy controls	BAI	AA more symptoms of anxiety than healthy controls, no difference with psoriasis	20^3^
Sayar et al. (69)	Turkey	NR	31	100	23.8 (2.5)	NR	40 healthy controls	STAI	AA more state and trait anxiety	55^3^
Sellami et al. (70)	Tunisia	March–July 2010	50	48	32.92 (11.81)	NR	50 healthy controls	HADS	AA more symptoms of anxiety than healthy controls	45^3^
Senna et al. (71)	USA	January 2011–December 2018	68,121	39	40.3 (17.8)	98.1% AA, 1.3% AT, 0.6% AU	No	ICD-9 and ICD-10 codes	8.4% had an anxiety disorder	45.83^4^
Sorour et al. (14)	Egypt	NR	208	58.65	NR	NR	1,042 dermatology patients	DSM-5 interview	19.71% of AA had anxiety diagnosis, no effect of gender. No difference with psoriasis. Less symptoms than acne, vitiligo, urticaria, and atopic dermatitis.	55^3^
Tan et al. (72)	China	December 2012–August 2013	168	50	34.5 (11.5)	88.1% AA, 11.9% AT/AU	100 healthy controls	SCL-90-R	AA more symptoms of anxiety and phobic anxiety than controls	41.67^5^
Titeca et al. (22)	13 European countries	37	NR	NR	NR	NR	1,359 healthy controls, 20 AGA	HADS	AA more symptoms of anxiety than healthy controls and AGA	70^3^
Tzur Bitan et al. (15)	Israel	2018	41,055	62.9	39.97 (13.61)	NR	41,055 healthy controls	ICD-9 codes	AA higher risk of anxiety disorder than controls	80^3^
Willemsen et al. (73)	Belgium	September 2006–August 2009	21	24	41.95 (13.79)	33% patchy AA, 14% ophiasis, 29% AT, 24% AU	No	SCL-90	AA more symptoms of anxiety than norm group	54.17^7^
Willemsen et al. (74)	Belgium	April 1999–April 2004	28	35.71	33.4	21.43% AA, 21.43% ophiasis, 28.57% AU, 3.57% AT	No	SCL-90	AA more symptoms of anxiety than norm group	50.00^7^
Yoon et al. (75)	South Korea	January 2015–February 2016	1,203	52.12	39.45 (12.21)	NR	No	BAI	10.1% symptoms of anxiety, 4.2% severe symptoms of anxiety	41.67^4^
Yu et al. (76)	China	October 2013–December 2014	130	41.5	31.78 (10.34)	NR	212 AGA	S-AS	No significant differences	70^3^

AA, alopecia areata; AGA, alopecia androgenetica; AU, alopecia universalis; AT, alopecia totalis; NR, not reported; PsA, psoriatic arthritis; SD, seborrheic dermatitis; TE, telogen effluvium; TV, tinea versicolor.

^1^Some patients had multiple episodes, with different forms of alopecia. Hence, the total is higher than 100%.

^2^This questionnaire was not administered to the control group.

^3^As measured by the NIH Quality Assessment of Case-Control studies.

^4^As measured by the NIH Quality Assessment Tool for Observational Cohort and Cross-Sectional Studies.

^5^As measured by the QAVALS (26).

^6^As measured by the NIH Quality Assessment of Controlled Intervention Studies.

^7^As measured by the NIH Quality Assessment Tool for Before-After (Pre-Post) Studies with no Control Group.

#### Children and adults

Three studies with a total of 5,665 patients with AA, reported that people with AA experienced more symptoms of anxiety and were diagnosed with anxiety more often than healthy controls (32–34). One smaller study (*n* = 24) (35) did not find a difference in the amount of symptoms of anxiety between people with AA and healthy controls.

When people with alopecia were compared to people with another (dermatological) condition, studies found that people with AA were diagnosed with an anxiety disorder more often than other hospitalized patients in general (34), but no differences were found for people with vitiligo (33).

One study without a control group (36) found that 19.1% of the adults with alopecia reported heightened symptoms of anxiety or depression. The same study also reported that 34.1% did not experience any symptoms of anxiety or depression. In the other study without a control group 89.0% of people with alopecia reported little to no symptoms of anxiety (38). Around 8% of people with AA reported moderate symptoms of anxiety.

#### Children

Of the papers investigating anxiety disorders, one study (46) reported that over half of the children had an anxiety disorder. However, this study included only 12 children and used the DSM-III-R, which was published in 1987. Two other studies reported that 7.1–16% had a generalized anxiety disorder, 7.1–8% had a separation anxiety disorder and 28.6% had a specific phobia (40, 44). However, none of the studies specified the number of patients with more than one anxiety disorder. It remains unclear from this data how many children with AA are diagnosed with an anxiety disorder. In a study by Altunisik et al. (39), 51.8% of the children was diagnosed with at least one anxiety disorder. This did not differ significantly from children with another dermatological condition.

When looking at symptoms of anxiety, studies comparing children with AA to healthy controls found mixed results. On the one hand, Bilgiç et al. (41) reported more state and trait anxiety in children aged 8–12 with AA. They did not find any differences for adolescents aged 12–18. On the other hand, Díaz-Atienza et al. (42) did not find significant differences when comparing children with AA to their siblings. Erdoğan et al. (43) found no difference on the Beck Anxiety Inventory (BAI), but found more child-reported separation anxiety and total anxiety and parent-reported panic disorder and total anxiety than healthy controls on the Revised Child Anxiety and Depression Scales (RCADS).

Studies comparing children with AA to children with other (dermatological) conditions found no differences in symptoms of anxiety when comparing to other dermatological conditions (39), epilepsy (42) and vitiligo (43). Liakopoulou et al. (45) found that children scored higher on worry, oversensitivity, and concentration than other patients.

#### Adults

Eight papers studied the prevalence of anxiety disorders in adults with AA (*n* = 86,014). These studies reported point prevalence rates of 3.24% (64), 4% (17), 8.4% (71), and 13.70% (58). Several papers also reported that people with AA have a higher chance of being diagnosed with an anxiety disorder in comparison to healthy controls (13, 15, 17, 64). Prevalence rates of specific anxiety disorders in people with AA range from 7.4% for specific phobias and 22.2–39% for generalized anxiety disorders (57, 66). The lifetime prevalence of specific phobia and panic disorder was estimated at 23 and 13%, respectively (57).

**FIGURE 2:**
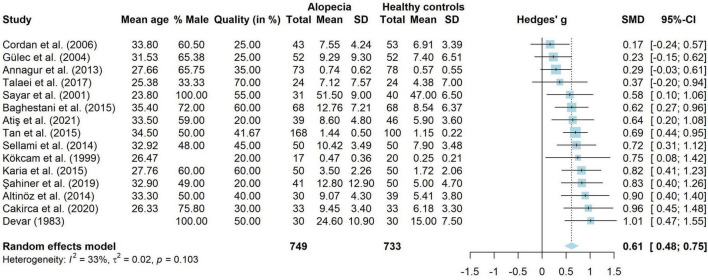
Forest plot for symptoms of anxiety in adults with alopecia compared to healthy controls.

When looking at symptoms of anxiety, 15 studies compared people with AA (*n* = 749) to healthy controls (*n* = 733). These results were combined in a meta-analysis, shown in Figure 2. The results showed that adults with AA reported significantly more symptoms of anxiety than people without AA (*g* = 0.61, 95% CI [0.48, 0.75], *p* < 0.001), with a medium to large effect. There was little heterogeneity (*I*^2^ = 33.1%, 95% CI [<0.01, 64.0], τ^2^ = 0.02, 95% CI [<0.01, 0.12]) and visual inspection of the funnel plot showed no indication for publication bias. Egger’s test also did not show indications for a publication bias [*t*(13) = 0.94, *p* = 0.363]. Thirteen studies without missing data were included in a meta-regression. The model did not explain any variance in the effect sizes (*R*^2^ = <0.01%), with a residual heterogeneity of *I*^2^ = 46.59%. Mean age (*g* = 0.01, *p* = 0.802, 95% CI [−0.04 to 0.05]), percentage male (*g* = <−0.01, *p* = 0.858, 95% CI [−0.01 to 0.01]) and quality score (*g* = <0.01, *p* = 0.583, 95% CI [<−0.01 to 0.01]) did not influence study effect sizes.

Studies comparing people with AA to people with other (dermatological) conditions showed mixed results. For the majority of studies, no significant differences were found. For instance, no differences were found when comparing to people with chronic urticaria (49), vitiligo (19, 17), seborrheic dermatitis (58), tinea versicolor (60), alopecia androgenetica and telogen effluvium (67), and psoriasis (68, 14). A smaller number of studies reported that adults with AA experienced more symptoms of anxiety than patients with benign skin lesions (54) and alopecia androgenetica (22, 76), but less than people with psoriasis (17), acne, vitiligo, chronic urticaria, and atopic dermatitis (14).

Three studies compared adults with AA (*n* = 61) to a norm group. These studies all reported more symptoms of anxiety in adults with AA (73, 74, 77).

### Depression

The results for depression are shown in Table 2.

**TABLE 2:** Results for depression.

References	Country	Year	*N*	% male	Age (*M*, SD)	% AA, AT, AU	Controls	Measures	Conclusions	Quality score (%)
**Pediatric and adult samples**										
Ataseven et al. (32)	Turkey	NR	43	72.1	23.42 (11.41)	NR	30 healthy controls	HAM-D; CDI	More symptoms of depression in AA compared to controls	30^1^
Chu et al. (37)	Taiwan	2000–2009	5,117	49.2	NR	NR	20,468 healthy controls	ICD-9 codes	2.9% AA has depression diagnosis, more often than controls	80^1^
Ghajarzadeh et al. (78)	Iran	January 2009–January 2010	100	69	23.02 (33.4)	NR	100 psoriasis, 100 vitiligo	BDI	No difference AA and psoriasis/vitiligo	55^1^
Gutierrez et al. (79)	USA	2006–2016	2,298,432 visits to dermatologist	35	37.8 (18.04)	NR	No	ICD-9 and ICD-10 codes	4.3% of the visits was related to depression	58.33^2^
Jagtiani et al. (80)	India	NR	38	65.8	25.79 (8.82)	NR	80 AV, 56 psoriasis	BDI	AA not significantly different from patients with acne vulgaris or psoriasis	55^1^
Kökcam et al. (33)	Turkey	NR	17	NR	26.47 (12.2)	NR	11 vitiligo, 20 healthy controls	SCL-90-R; ZSDS	AA more symptoms of depression than healthy controls, no difference with vitiligo	20^1^
Laitinen et al. (81)	Finland	1987–2016	176	25	29.7 (NR)	NR	No	ICD-9 and ICD-10 codes	3.98% was diagnosed with depression	54.17^2^
Layegh et al. (82)	Iran	October 2005–May 2006	73	NR	NR	NR	78 AV, 62 psoriasis, 87 vitiligo	BDI	31.51% minor depression, 23.29% mild depression, 24.66% moderate depression, 20.55% severe depression	55^1^
Liu et al. (83)	USA	NR	91 children, 292 adults	Child: 34.4%, adult: 27.9%	Child: 10 (2.92), adult: 41 (15.3)	NR	No	PHQ-9	On average mild symptoms of depression in children and adults	20.83^2^
Marahatta et al. (38)	Nepal	August 2015–July 2016	75	53.3	29.40 (9.90)	NR	No	BDI	66.7% depressive complaints. No relation to disease severity.	45.83^2^
Singam et al. (34)	USA	2002–2012	5,605 hospitalized patients	38.3	42.2 (NR)	NR	Hospitalized patients without AA (N unknown)	ICD-9 codes	AA more mood disorders than controls	45^1^
Talaei et al. (35)	Iran	April–July 2005	24	33.33	25.38 (8.32)	NR	24 healthy controls	SCL-90-R	No difference AA and controls	70^1^
Vallerand et al. (84)	GB	NR	6,861	43.9	32.20 (13.50)	NR	6,137,342 healthy controls	Read codes	AA higher chance of depression than controls	60^1^
Vélez-Muñiz et al. (36)	Mexico	March 2017–February 2018	32 children, 94 adults	41	NR	92.9% patchy AA, 3.2% AT, 1.6% ophiasis, 1.6% AU	No	DSRS-C; HADS	Children: 6.3% symptoms of depression. Adults: 19.1% subclinical depression or anxiety, 34.1% no symptoms of anxiety or depression.	50^2^
**Pediatric samples**										
Altunisik et al. (39)	Turkey	NR	27	29.6	11.9 (3.3)	85.19% AA, 14.81% AU	30 dermatology patients	K-SADS-PL; CDI	No difference AA and controls. 14.8% symptoms of depression.	65^1^
Andreoli et al. (40)	Italy	1997–2000	176	NR	NR	NR	No	Diagnosis by psychologist	10% dysthymia	25^2^
Bilgiç et al. (41)	Turkey	NR	74	55.41	12.1 (2.8)	NR	65 healthy controls	CDI	AA more symptoms of depression than controls	65^1^
Conic et al. (85)	USA	2019	3,510	44.7	26.2% <10 years, 73.8% 10–18 years	NR	8,310,710 patients without AA	Diagnoses in patient file	AA diagnosed with depression (2.6%) more often than controls (0.6%)	10^1^
Díaz-Atienza and Gurpegui (42)	Spain	NR	31	52	12.2 (3.8)	51.61% AA, 48.39% AU/AT	23 epilepsy, 25 siblings	CDI	No differences AA and epilepsy or siblings	65^1^
Erdoğan and Gür (43)	Turkey	October 2018–December 2019	31	54.83	12.54 (3.56)	100% AA	29 vitiligo, 30 healthy controls	RCADS-C; RCADS-P	AA more depression than healthy controls, no difference vitiligo	60^1^
Ghanizadeh (44)	Iran	August 2004–November 2006	14	NR	11.66 (6.08)	NR	No	K-SADS-PL	50% has diagnosis of depression	50^2^
Liakopoulou et al. (45)	Greece	NR	33	30.3	10.5 (0.3)	NR	30 patients from pediatrician	CDI	No difference AA and controls	40^1^
Reeve et al. (46)	USA	NR	12	NR	11.5 (2.9)	NR	No	DICA-R; CDS	No heightened group average	37.5^2^
**Adult samples**										
Aghaei et al. (47)	Iran	NR	40	44.8	35.2 (9.2)	NR	40 healthy controls	BDI	AA more symptoms of depression than controls	35^1^
Alfani et al. (48)	Italy	November 2009–October 2010	73	45.2	25.2 (9.2)	61.7% AA, 26.0% AT, 12.3% AU	73 healthy controls	MMPI-2	AA patients score above cut-off for depre ssion more often than controls	35^1^
Altinöz et al. (49)	Turkey	September 2011–October 2012	30	50	33.3 (8.9)	NR	30 urticaria, 39 healthy controls	HADS	AA more symptoms of depression than healthy controls. No difference with urticaria.	40^1^
Annagur et al. (50)	Turkey	NR	73	65.75	27.66 (7.79)	100% AA	78 healthy controls	SCL-90	AA more symptoms of depression than controls	35^1^
Atış et al. (19)	Turkey	NR	39	59	33.5 (11.6)	NR	46 vitiligo, 46 healthy controls	HADS	No differences between AA, vitiligo and healthy controls	20^1^
Baghestani et al. (51)	Iran	NR	68	72	35.4 (7.6)	100% AA	68 healthy controls	HAM-D	AA more symptoms of depression than controls (OR = 4.48)	60^1^
Bain et al. (52)	UK	NR	39	23.07	43.15 (12.43)	NR	23 PsA; 26 healthy controls	HADS*	Depressive symptoms in 18%. Less severe symptoms with higher SALT scores.	30^1^
Balieva et al. (53)	13 European countries	November 2011–February 2013	33	33.3	42.8 (14.1)	NR	1,359 healthy controls	EQ-5D-3L	AA 4 times higher chance of anxiety/depression than controls	65^1^
Bashir et al. (54)	Pakistan	January–March 2007	3	NR	NR	NR	No	GHQ-12; interview	1 person was diagnosed with depression	41.67^2^
Bukharia and Jain (55)	India	NR	100	48	54% 15–30 years, 46% 31–50 years	NR	100 TE, 100 healthy controls	HAM-D	23.68% AA and 33.33% TE with symptoms of depression	45^2^
Cakirca et al. (56)	Turkey	March–December 2017	33	75.8	26.33 (6.08)	NR	33 healthy controls	HADS	AA more depressive symptoms than controls	30^1^
Colon et al. (57)	USA	April 1985–October 1987	31	29	35.70 (10.23)	74% AA, 23% AT, 42% AU^3^	No	DIS	Lifetime prevalence depression 39%, dysthymia 16%	33.33^2^
Conic et al. (58)	USA	2005–2014	584	31.5	35.54 (19.28)	94.7% AA, 2.05% AT, 3.25% AU	172 SD	Diagnoses in patient file	No difference with control group	35^1^
Cordan Yazici et al. (59)	Turkey	NR	43	60.5	33.80 (10.02)	95.35% AA, 4.65% AT	53 healthy controls	HADS	No difference with controls	25^1^
Dai et al. (90)	Taiwan	NR	2,123	44.8	31.39 (9.02)	NR	2,298 siblings, 9,192 healthy controls	ICD-9 codes	7.87% of AA with MDD diagnoses, 8.22 times higher chance than healthy control. A total of 2.55 higher chance than siblings.	85^1^
Devar (60)	India	NR	30	100	NR	NR	30 TV, 30 healthy controls	BDI	AA more symptoms of depression than healthy controls, no difference with TV	50^1^
Endo et al. (61)	Japan	June 2009–August 2010	122	33.1	38.3 (16.5)	NR	No	CES-D	AA more symptoms of depression than norm group	56.25^4^
Gallo et al. (77)	Italy	NR	16	37.5	45.95 (13.25)	NR	No	BSI	AA more symptoms of depression than norm group	39.29^5^
Güleç et al. (62)	Turkey	March 2001–January 2002	52	65.38	31.53 (12.61)	94.23% AA, 3.65% AU, 1.92% AT	52 healthy controls	BDI	No differences between AA and controls	25^1^
Gupta and Gupta (16)	USA	NR	45	24.44	44.7 (11.6)	NR	72 AV, 146 AD, 217 psoriasis	CRSD	AA less depressive symptoms than AV and psoriasis, no difference with AD	15^1^
Karia et al. (17)	India	NR	50	66.00	27.76 (NR)	NR	50 psoriasis, 50 healthy controls	DSM-IV-TR diagnosis	18% AA depression diagnoses. More often than healthy controls, less often than psoriasis.	60^1^
Kim et al. (13)	South Korea	2002–2013	7,706	51.9	54.6% 20–39, 39.4% 40–59, 6.1% 60+	NR	30,824 people without AA	ICD-10 codes	AA higher chance of depression than controls	65^1^
Kose et al. (63)	Turkey	NR	18	100	21.3 (NR)	NR	No	BDI	On average subclinical depressive symptoms	50.00^6^
Macbeth et al. (64)	UK	January 2009–December 2018	5,435	45.9	38.93 (14.35)	NR	21,470 healthy controls	Diagnoses in patient file	AA higher chance of depression than controls	80^1^
Mirza et al. (87)	USA	2002–2012	138	0	NR	NR	No	Diagnoses in patient file	21.74% has depression diagnosis	58.33^2^
Pascual-Sánchez et al. (88)	Spain	NR	16	0	45.1 (NR)	100% AU	No	BDI	On average subclinical depressive symptoms	29.17^6^
Rajoo et al. (65)	Australia	NR	83	NR	40.95 (13.24)	NR	No	DASS-21	47.0% reported extreme depressive symptoms	54.17^2^
Ruiz-Doblado et al. (66)	Spain	NR	32	15	NR	NR	No	SCAN	7.4% depression diagnosis, 7.4% previously diagnosed, but currently free of symptoms	37.5^2^
Şahiner et al. (68)	Turkey	August 2009–July 2010	41	49	32.9 (10.5)	NR	30 psoriasis, 50 healthy controls	BDI	AA more depressive symptoms than healthy controls, no difference with psoriasis	20^1^
Sayar et al. (69)	Turkey	NR	31	100	23.8 (2.5)	NR	40 healthy controls	BDI	AA more symptoms of depression than controls	55^1^
Sellami et al. (70)	Tunisia	March–July 2010	50	48	32.92 (11.81)	NR	50 healthy controls	HADS	AA more symptoms of depression than controls	45^1^
Senna et al. (71)	USA	January 2011–December 2018	68,121	39	40.3 (17.8)	98.1% AA, 1.3% AT, 0.6% AU	No	ICD-9 and ICD-10 codes	9.5% had depression diagnosis	45.83^2^
Sorour et al. (14)	Egypt	NR	208	58.65	NR	NR	1,042 dermatology patients	DSM-5 interview	19.71% of AA had diagnosis of depression. 24.33% in psoriasis, 55.34% acne vulgaris, 31.47% vitiligo, 43.64% urticarial, and 43.63% in atopic dermatitis	55^1^
Tan et al. (72)	China	December 2012–August 2013	168	50	34.5 (11.5)	88.1% AA, 11.9% AT/AU	100 healthy controls	SCL-90-R	AA more symptoms of depression than controls	41.67^4^
Titeca et al. (22)	13 European countries	37	NR	NR	NR	NR	1,359 healthy controls, 20 AGA	HADS	AA more symptoms of depression than healthy controls	70^1^
Tzur Bitan et al. (15)	Israel	2018	41,055	62.9	39.97 (13.61)	NR	41,055 healthy controls	ICD-9 codes	AA diagnosed with depression more often than controls	80^1^
Willemsen et al. (73)	Belgium	September 2006–August 2009	21	24	41.95 (13.79)	33% patchy AA, 14% ophiasis, 29% AT, 24% AU	No	SCL-90	AA more symptoms of depression than norm group	54.17^6^
Willemsen et al. (74)	Belgium	April 1999–April 2004	28	35.71	33.4 (NR)	21.43% AA, 21.43% ophiasis, 28.57% AU, 3.57% AT	No	SCL-90	AA more symptoms of depression than norm group	50.00^6^
Yoon et al. (75)	South Korea	January 2015–February 2016	1,203	52.12	39.45 (12.21)	NR	No	BDI	40.9% depressive symptoms. Women more often than men, more symptoms with more severe AA.	41.67^2^
Yu et al. (76)	China	October 2013–December 2014	130	41.5	31.78 (10.34)	NR	212 AGA	ZSDS	No differences between AA and AGA	70^1^

*This questionnaire was not administered to the control group.

AD, atopic dermatitis; AGA, alopecia androgenetica; AV, acne vulgaris; PsA, psoriatic arthritis; SD, seborrheic dermatitis; TE, telogen effluvium; TV, tinea versicolor.

^1^As measured by the NIH Quality Assessment of Case-Control studies.

^2^As measured by the NIH Quality Assessment Tool for Observational Cohort and Cross-Sectional Studies.

^3^Some patients had multiple episodes, with different forms of alopecia. Hence, the total is higher than 100%.

^4^As measured by the QAVALS (26).

^5^As measured by the NIH Quality Assessment of Controlled Intervention Studies.

^6^As measured by the NIH Quality Assessment Tool for Before-After (Pre-Post) Studies with no Control Group.

#### Children and adults

In terms of diagnoses of depression, 4.3% of the visits to a psychologist by people with AA were related to depression (79). The point prevalence varied from 2.9% (37) to 3.98% (81). Different studies reported that people with AA were diagnosed with depressive disorders (37, 84) and mood disorders in general (34) significantly more often than healthy controls.

When looking at depressive symptoms, results concerning comparisons to healthy controls are mixed. Two studies, with a combined sample size of 60, reported more depressive symptoms in people with AA (32, 33), while one study did not find any significant differences (*n* = 24) (35).

Two studies compared people with AA to people with another (dermatological) condition. They did not find significant differences concerning the amount of depressive symptoms when comparing to people with psoriasis or vitiligo (78) or people with acne vulgaris, psoriasis or vitiligo (80).

Four studies (*n* = 657) did not use a control group. They found little to no depressive symptoms in 31.5% (82), 33.3% (38), and 34.1% (36) of people with AA. According to these studies around 60–65% of people with AA experience at least moderate depressive symptoms.

#### Children

Three studies (*n* = 3,700) investigated depressive disorders. A small study of 14 children found 50% of the children to be eligible for a diagnosis of depressive disorder (44). Bigger studies reported that 10% of the children were diagnosed with dysthymia (40) and that children with AA were diagnosed with a depressive disorder more often than other patients (85).

Three studies (*n* = 136) investigated symptoms of depression in comparison to healthy controls. Two studies found more depressive symptoms in children with AA (41, 43), while one study did not find a significant difference when comparing to unaffected siblings (42).

Four studies compared children with AA to children with a different (dermatological) condition. They did not find a difference in depressive symptoms when comparing children with AA to children with other dermatological conditions (39), epilepsy (42), vitiligo (43), and pediatric patients in general (45).

One study with 14 children with AA did not use a control group. This study did not find a heightened group average for depressive symptoms (46).

#### Adults

Several studies investigated the prevalence of depressive disorders in adults with AA. One study, conducted in the late 1990s, found a lifetime prevalence of 39% for depression and 16% for dysthymia (57). Estimates for point prevalence range from 7.4% (66), 9.5% (71), 18% (17), 21.74% (87) to 55.29% (14). The largest and most recent study found a point prevalence of 9.5% (71). Furthermore, adults with AA have a higher chance of being diagnosed with a depressive disorder than healthy controls (13, 15, 17, 64). One study did not find any difference in the number of diagnoses (58). There were no differences in the number of diagnoses when comparing to adults with psoriasis or vitiligo (17) or seborrheic dermatitis (58).

**FIGURE 3:**
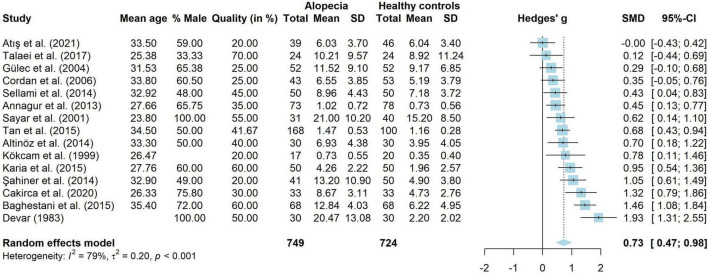
Forest plot for symptoms of depression in adults with alopecia compared to healthy controls.

Fifteen studies compared adults with AA to healthy controls on the amount of depressive symptoms. These studies were analyzed in a meta-analysis. The results are shown in Figure 3. A total of 749 adults with AA and 724 healthy controls were analyzed. Adults with AA reported significantly more depressive symptoms than the control group (*g* = 0.73, 95% CI [0.47, 0.98], *p* < 0.001), with a medium to large effect. There was considerable heterogeneity (*I*^2^ = 78.5%, 95% CI [65.2, 86.8], τ^2^ = 0.20, 95% CI [0.08, 0.62]). Visual inspection of the funnel plot showed no signs of publication bias and Egger’s test was not significant [*t*(13) = 0.80, *p* = 0.438]. Thirteen studies without missing data were included in a meta-regression. The model explained very little variance in the effect sizes (*R*^2^ = 1.21%) and residual heterogeneity was high (*I*^2^ = 77.27%). Mean age (*g* = 0.04, *p* = 0.289, 95% CI [−0.03 to 0.12]), percentage male (*g* = 0.01, *p* = 0.168, 95% CI [−0.01 to 0.03]) and quality score (*g* = 0.01, *p* = 0.265, 95% CI [−0.01 to 0.03]) did not influence study effect sizes.

Nine studies used a control group of adults with a different (dermatological) condition to assess the amount of depressive symptoms. The vast majority of the studies did not find any significant differences. For instance, no differences were found when comparing to chronic urticaria (49), vitiligo (19), telogen effluvium (55), tinea versicolor (60), atopic dermatitis (16), psoriasis (68), and alopecia androgenetica (22, 76). One study found that adults with AA reported less depressive symptoms than adults with acne vulgaris or psoriasis (16).

Studies without a control group found that people with AA (*n* = 183) reported more symptoms of depression than a norm group (61, 73, 74, 77). On average, they reported subclinical symptoms (63, 88). Estimates of the prevalence rates of people with depressive symptoms were 47.0% (65) and 40.9% (75).

### Quality of life

The results for QoL are shown in Table 3.

**TABLE 3:** Results for quality of life.

References	Country	Year	*N*	% male	Age (*M*, SD)	% AA, AT, AU	Controls	Measures	Conclusions	Quality score (%)
**Pediatric and adult samples**										
Ghajarzadeh et al. (78)	Iran	January 2009–January 2010	100	69	23.02 (33.4)	NR	100 psoriasis, 100 vitiligo	DLQI; SF-36	AA better QoL than psoriasis. No difference with vitiligo. On average moderate effect on QoL.	55^1^
Liu et al. (83)	USA	NR	91 children, 292 adults	Child: 34.4%; adult: 27.9%	Child: 10 (2.92); adult: 41 (15.3)	NR	No	CDLQI; DLQI; FDLQI	Children, adults, and family members have moderate effect on QoL. Worse QoL related to more depressive symptoms.	20.83^2^
Park et al. (92)	South Korea	NR	40	27.5	30.0% 10–19 years, 17.5% 20–29, 17.5% 30–39, 17.5% 40–49, 17.5% 50+	NR	No	Skindex-29	Symptoms, emotions, and total score very little impairment. Functioning mild impairment	37.5^2^
Vélez-Muñiz et al. (36)	Mexico	March 2017–February 2018	32 children, 94 adults	41	NR	92.9% patchy AA, 3.2% AT, 1.6% ophiasis, 1.6% AU	No	CDLQI; DLQI	Children small impairment on QoL. Adults moderate effect. No differences for gender, disease duration, and disease severity.	50^2^
**Pediatric samples**										
Bilgiç et al. (41)	Turkey	NR	74	55.41	12.1 (2.8)	NR	65 healthy controls	PedsQL-P; PedsQL-C	Less QoL on child and parent reports. Less psychosocial QoL on parent reports.	65^1^
Erdoğan and Gür (43)	Turkey	October 2018–December 2019	31	54.83	12.54 (3.56)	100% AA	30 healthy controls; 29 vitiligo	CDLQI	AA worse QoL than vitiligo	60^1^
Putterman et al. (93)	USA	April 2017–July 2018	153	43.79	11.0 (4.8)	NR	No	CDLQI; FDLQI; QLCCDQ	On average small effect on child QoL, moderate effect for family members. Worse QoL for more disease severity and worse emotional QoL for higher age.	50^2^
**Adult samples**										
Abedini et al. (94)	Iran	October 2013–October 2014	176	64.23	31.39 (9.05)	NR	No	DLQI	Patients with mild AA moderate effect on QoL, patients with severe AA very large effect on QoL.**** Patients with more severe AA reported worse QoL on: symptoms and feelings, daily activities, leisure, personal relationships, work and school, treatment, and the total score.	50^2^
Abideen et al. (95)	India	NR	60	65	33.9 (9.3)	NR	No	DLQI	30% no effect on QoL, 55% small effect, 6.7% moderate effect, 8.3% very large effect	20.83^2^
Al-Mutairi and Eldin (91)	Kuwait	August 2002–July 2009	2,962 (300 for DLQI)	65.02	58.03% between 21 and 40 years	NR	300 healthy controls	DLQI	No difference between males and females or disease duration. Worse QOL for more severe alopecia	40^1^
Andersen et al. (20)	Denmark	NR	1,494	33	51.3 (16.0)	NR	No	DLQI; EQ-5D-5L	75% no effect on QoL. On average small effect.	41.67^2^
Atış et al. (19)	Turkey	NR	39	59	33.5 (11.6)	NR	46 healthy controls, 46 vitiligo	DLQI	On average moderate effect, no difference with vitiligo	20^1^
Balieva et al. (53)	13 European countries	November 2011–February 2013	33	33.3	42.8 (14.1)	NR	1,359 healthy controls	EQ-5D-3L	No significant difference for mobility, self-care, activity, and pain/discomfort	65^1^
de Hollanda et al. (96)	Brazil	January 2011–October 2012	37	37.84	35.89 (11.59)	NR	49 healthy controls	SF-36	AA score lower on mental health, role emotional and social functioning. No differences for vitality, bodily pain, general health, physical functioning, and role physical.	55^1^
Dubois et al. (97)	France	NR	60	35.00	40.1 (15.2)	NR	Dermatologic conditions and healthy controls from literature	SF-36; Skindex	Lower scores on role-physical, general health, vitality, social functioning, role-emotional, and mental health	41.67^2^
Endo et al. (61)	Japan	June 2009–August 2010	122	33.1	38.3 (16.5)	NR	No	SF-8	Average scores on physical and mental functioning	56.25^3^
Essa et al. (98)	Egypt	January–June 2015	17	NR	NR	NR	500 healthy controls	Skindex-16	No difference AA and dermatological conditions. AA worse QoL than healthy controls.	41.67^3^
Fayed et al. (99)	Egypt	February 2015–January 2016	41	78	26.68 (4.49)	NR	No	DLQI	0% no effect on QoL, 4.9% small effect, 29.3% mild effect, 29.3% moderate effect, 36.6% very large effect	50^4^
Gonul et al. (21)	Turkey	NR	56	55.4	29.34 (8.13)	92.86% AA, 7.14% AT	82 AGA	Hairdex; TQL	AA better QoL than AGA on total, emotions, functions, symptoms, and self-confidence. No difference on stigmatization and TQL.	60^1^
Güleç et al. (62)	Turkey	March 2001–January 2002	52	65.38	31.53 (12.61)	94.23% AA, 3.65% AU, 1.92% AT	52 healthy controls	SF-36	AA worse QoL on vitality and mental health than controls. AA higher QoL than healthy controls on social functioning.	25^1^
Han et al. (100)	USA	August 2018–November 2019	141	26.2	43.3 (15.6)	76.6% AA, 13.5% AU, 9.9% AT	No	AASIS	More stress is related to lower QoL	41.67^2^
Jankovic et al. (101)	Serbia	April 2012–June 2013	60	26.7	37.35 (14.26)	NR	110 psoriasis, 66 AD; 140 OM	DLQI; SF-36; Skindex-29	AA better QoL than psoriasis. Partially better QoL than AD and OM.	41.67^2^
Karia et al. (17)	India	NR	50	66	27.76 (NR)	NR	50 psoriasis, 50 healthy controls	WHOQOL-BREF	AA higher QoL than psoriasis and healthy controls	60^1^
Lai et al. (102)	Australia	NR	36	19.4	41 (14.5)	41.7% patchy, 25.0% AT, 33.3% AU	No	AASIS; aQoL-8D	No difference with norm group	75^5^
Liu et al. (103)	USA	NR	30	53.3	38.00 (21.80)	NR	No	Skindex-16	No difference between males and females	29.17^4^
Masmoudi et al. (104)	Tunisia	March–July 2010	50	48	32.92 (11.81)	NR	50 healthy controls	SF-36	AA worse scores on mental health, role emotional, social functioning, general health and total mean score. No significant differences for physical functioning, role physical and bodily pain. No relation QoL and disease severity.	55^1^
Nasimi et al. (105)	Iran	August 2017–August 2018	100	65	29.24 (8.31)	NR	No	AA-QLI; DLQI	On average very large effect. Males better QoL than females.	20.83^3^
Nijsten et al. (106)	Italy	NR	46	NR	NR	NR	151 AV; 76 psoriasis, 54 SD; 27 vitiligo; 100 nevi	Skindex-29	17.4% in worst category for total, 21.7% in worst category for emotions	55^1^
Öztürkcan et al. (107)	Turkey	January–February 2004	3	NR	NR	NR	16 CD, 6 psoriasis, 3 urticaria, 16 TP; 35 AV	DLQI	On average small effect	41.67^3^
Qi et al. (108)	China	January 2010–July 2012	698	50	38.8 (12.0)	82.5% patchy, 17.5% AT/AU	No	DLQI	On average moderate effect on QoL	54.17^2^
Reid et al. (109)	USA	March–November 2009	23	0	NR	NR	33 TE, 41 AGA, 7 unknown alopecia	Skindex-16	No differences on QoL for different alopecia types	55^1^
Russo et al. (67)	Italy	September 2016–September 2017	27	33.3	37.55 (10.37)	NR	80 AGA, 36 TE	DLQI	Females worse QoL than men. No differences with AGA or TE.	50^1^
Sampogna et al. (110)	Italy	NR	5	NR	NR	NR	Dermatological conditions	Scalpdex; Skindex-29	Average impact on symptoms, emotions, and functioning	55^1^
Sanclemente et al. (111)	Colombia	NR	11	NR	NR	NR	Dermatological conditions	Skindex-29	Median score indicates moderate effect on total score, symptoms, emotions, and functioning	60^1^
Senna et al. (71)	USA	2019	259	49.4	39.1 (13.6)	NR	No	Skindex-16	Worse QoL for longer disease duration, higher disease severity, and females	41.67^2^
Temel et al. (112)	Turkey	NR	50	46	30.92 (10.92)	84% AA, 6% AT, 10% AU	50 AV; 50 vitiligo	DLQI	On average moderate effect on QoL. No difference with AV or vitiligo.	40^1^
Titeca et al. (22)	13 European countries	37	NR	NR	NR	NR	1,359 healthy controls, 20 AGA	DLQI	Worse QoL than AGA	70^1^
Willemse et al. (113)	Multiple countries	NR	243	11	37.9 (13.0)	NR	No	DLQI	On average no effect on QoL. No differences for gender or disease severity. Worse QoL for shorter disease duration.	54.17^2^
Willemsen et al. (73)	Belgium	September 2006–August 2009	21	24	41.95 (13.79)	33% patchy, 14% ophiasis, 29% AT, 24% AU	No	SF-36; Skindex-17	SF-36: average physical functioning, below average mental functioning in comparison to norm group**** Skindex: moderate effect on psychosocial functioning, less physical symptoms in comparison to norm group	54.17^4^
Yoon et al. (75)	South Korea	January 2015–February 2016	1,203	52.12	39.45 (12.21)	NR	No	Skindex-29	30.3% impaired QoL, 9.9% severely impaired QoL. Females worse QoL than men.	41.67^2^
Yu et al. (76)	China	October 2013–December 2014	130	41.5	31.78 (10.34)	NR	212 AGA	DLQI	AA worse QoL than AGA on total, symptoms and feelings, daily activities, and leisure. AA scored higher on treatment. No differences for work and school and personal relationships.	70^1^
**Unknown age**										
Jun et al. (116)	South Korea	March 2012–February 2017	161	NR	NR	NR	380 AGA	Skindex-29	AA worse scores than AGA on functioning and better scores on symptoms. No differences for emotions and total score.	45^1^
Shi et al. (114)	USA	NR	532	27	50% older than 40	52% AU/AT, 46.6% with 100% hair loss	No	DLQI; Skindex-16	On average moderate effect on QoL. Moderate effect on emotions, symptoms, and functioning	20.83^2^

AD, atopic dermatitis; AGA, alopecia androgenetica; AV, acne vulgaris; CD, contact dermatitis; HS, hidradenitis suppurativa; NF1, neurofibromatosis, type 1; NR, not reported; OM, onychomycosis; SD, seborrheic dermatitis; TE, telogen effluvium; TP, tinea pedis.

^1^As measured by the NIH Quality Assessment of Case-Control studies.

^2^As measured by the NIH Quality Assessment Tool for Observational Cohort and Cross-Sectional Studies.

^3^As measured by the QAVALS.

^4^As measured by the NIH Quality Assessment Tool for Before-After (Pre-Post) Studies with no Control Group.

^5^As measured by the NIH Quality Assessment of Controlled Intervention Studies.

#### Children and adults

People with AA reported worse QoL than people with psoriasis, but there was no difference with vitiligo (78). On average, people reported a small (36, 92) or moderate (36, 78, 83) impact on their QoL.

#### Children

Children with AA reported more impaired QoL than healthy controls (41, 43). In a study without a control group children with AA reported a small effect on their QoL (93).

#### Adults

**FIGURE 4:**
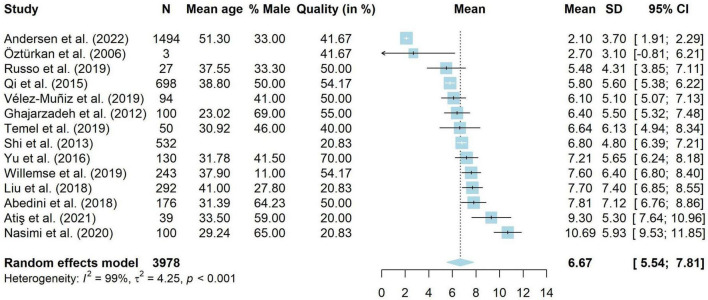
Forest plot for mean DLQI scores in adults with alopecia areata.

Fourteen studies used the Dermatology Life Quality Index (DLQI) to assess disease-specific QoL in 3,978 adults with AA (19, 20, 36, 67, 76, 78, 83, 94, 105, 107, 108, 112–114). These studies were included in a meta-analysis, shown in Figure 4. The total scores of the DLQI can be interpreted as follows: 0–1 = no effect on patient’s life, 2–5 = small effect, 6–10 = moderate effect, 11–20 = very large effect, 21–30 extremely large effect (115). Results from the meta-analysis showed that people with AA reported a weighted average of 6.67 (95% CI [5.54, 7.81]), which is a moderate effect. However, there was very high heterogeneity amongst studies (*I*^2^ = 98.9%, 95% CI [98.5, 99.0], τ^2^ = 4.25, 95% CI [2.07, 12.29], *p* < 0.001). Results should thus be interpreted with extreme caution. Meta-regressions were run on 11 studies without missing data. The model explained 62.89% of the variance in the data, but still included a substantial amount of heterogeneity (*I*^2^ = 89.56%). Mean age was negatively related to DLQI scores (*g* = −0.28, *p* = < 0.001, 95% CI [−0.44 to −0.12]). Studies with a higher mean age had lower DLQI scores and thus less impaired QoL. The same was true for the quality ratings of studies (*g* = −0.08, *p* = 0.009, 95% CI [−0.13 to −0.02]), where studies with a lower quality rating reported higher DLQI levels. The percentage male (*g* = −0.04, *p* = 0.200, 95% CI [−0.11 to 0.02]) was not significantly related to DLQI scores.

As two studies did not provide clear data on their sample and may have included children (78, 114), we conducted a sensitivity analysis to assess whether this influenced the results. Twelve studies (19, 20, 36, 67, 76, 83, 94, 105, 107, 108, 112, 113) with 3,346 people were included. The mean DLQI was unchanged (*M* = 6.68, 95% CI [5.33, 8.02]) and heterogeneity remained high (*I*^2^ = 98.8%, 95% [98.4, 99.0], τ^2^ = 5.14, 95% [2.38, 16.35]).

Five studies compared 188 adults with AA to healthy controls (53, 62, 96, 98, 104). There seemed to be no difference on physical functioning (53, 96, 104). However, adults with AA had more impaired mental (62, 96, 104) and overall (98) functioning.

Thirteen studies compared adults with AA to adults with another (dermatological) diagnosis (17, 20–22, 67, 76, 97, 101, 106, 109–112) and found very mixed results. On the one hand, no differences were found when comparing to adults with vitiligo (20, 112), alopecia androgenetica and telogen effluvium (67) and acne vulgaris (112). On the other hand, people with AA reported better QoL than people with psoriasis (101) or alopecia androgenetica (21). In yet other studies, people with AA reported worse QoL than people with alopecia androgenetica (22, 76).

#### Unknown samples

Two studies did not report whether they studied children or adults (114, 116). They found a moderate impact on QoL (114). When comparing to alopecia androgenetica, people with AA scored higher on subscales functioning and lower on symptoms (116). No differences were found for emotions and total score.

## Discussion

In this systematic review and meta-analysis we aimed to provide an overview of the current literature on anxiety, depression and QoL in people with AA. Results showed that people with AA experienced adverse psychosocial consequences in all three domains. Results also point to more diagnoses of anxiety and depression, as well as more symptoms of anxiety and depression, compared to healthy controls.

Meta-analytic results showed that people with AA experience more symptoms of anxiety and depression than healthy controls. With a medium to large effect for both meta-analyses, we can conclude that this constitutes a clinically relevant effect. Our results were unable to shed light on which patients are at risk for experiencing symptoms of anxiety or depression as average age, percentage male and quality of the studies did not explain variance in the effect sizes. While the same studies were included in both meta-analyses, we found high heterogeneity for depression but not for anxiety. The range for effect sizes is much larger in depression than anxiety, however it is unclear where this originates from.

Meta-analytic results also showed that people with AA experience a moderate impact on their QoL. We were able to include around 3,800 patients in this meta-analysis, which makes it likely that our results generalize to other adults with AA. However, as we found very high heterogeneity, the moderate impact of AA on QoL is unlikely to be true for everyone with AA. Subgroups may exist based on variables that were not studied in the current meta-analysis, such as severity of disease, medication use, duration of disease or other variables.

Results concerning people with AA compared to people with other dermatological diagnoses were mixed for anxiety, depression, and QoL. However, the majority of the studies seems to point to people with AA experiencing the same amount of anxiety, depression, and impairment of QoL as people with other diagnoses. So, even though patients with AA do not experience physical symptoms that people with other dermatological diagnoses may experience, such as pain or itching (117), their QoL is comparable.

While we did not directly compare age groups, some observations can be noted. Firstly, for all three domains more studies were included for adults than for children. Hence, conclusions for adults can be made with more certainty. Both for anxiety and depression results of children with AA compared to healthy controls were mixed, while results for adults showed that adults with AA experienced more symptoms of anxiety and depression than healthy controls. As the mean age of the studies with children was 11.85, it is possible that symptoms of anxiety do not develop before puberty or adulthood, when appearance and peer relations become more important. This is corroborated by other studies showing more appearance-related distress in puberty (118). For QoL only three studies were found for children, so direct comparisons are hard to make.

Overall, our results are in line with a previous meta-analysis finding positive associations between AA and experiencing (symptoms of) anxiety or depression (12). In addition, we have shown that adults with AA experience more symptoms of anxiety and depression than healthy controls. Our results concerning QoL are also in line with Toussi et al. (23), who found diminished QoL in children and adults with AA. More specifically, we found diminished QoL in mental wellbeing but not necessarily in physical wellbeing. This is slightly unsurprising, as AA is associated with little physical impairment. Despite this, qualitative studies have shown that losing one’s hair has a considerable impact on mental health (7, 8).

It is also noteworthy that many studies included patients that were referred to a dermatologist. This could introduce a selection bias, where those who experience less psychological complaints are less likely to visit a dermatologist. However, large studies on primary care databases also reiterate that patients with alopecia are diagnosed with anxiety and depression more often than patients without alopecia (64, 84), with a diagnosis of depression preceding the diagnosis of AA for some patients (84).

This systematic review also has some strengths and limitations. A particular strength is the thorough literature search conducted. A formal search was created by a librarian, yielding 1,249 unique records. With this thorough search it is highly likely that no relevant articles were missed in the search process.

Despite the thorough literature search, we could only include a limited amount of studies in a quantitative analysis. For instance, we did not have enough data to disentangle psychological wellbeing in separate forms of AA (i.e., areata, universalis, or totalis) or how psychological wellbeing was related to disease severity or disease duration. Another limitation is that the included studies did not look at the remitting and relapsing course of AA specifically. Most studies were cross-sectional and longitudinal studies were often designed to look at a medical or psychological intervention. Qualitative research has highlighted that the unpredictable nature of AA can lead to feelings of anger or stress (119), but this has not been studied quantitatively. Hence, it remains unclear how the remitting and relapsing course of AA influences psychological wellbeing. A third limitation is that the included studies did not provide data on medication use. Inclusion criteria were often unclear when it came to participants’ medication use and medication use was often omitted from reporting in the outcome data. We do know that medical treatments often fail to provide sustained hair regrowth and may lead to substantial side effects (3). Hence, it remains unclear whether the pros of medication use outweigh the cons.

Another limitation is that the goal of the included studies did not always line up with the goal of the current systematic review. For instance, this review also included questionnaire validation studies (107) and baseline data of randomized controlled trials (88). The data was therefore approached in a different manner than the original authors intended. This may impede the strength of the current conclusions. However, as the intention of this review was to provide a thorough overview of the current literature, minimal limitations were set for the inclusion of different types of papers.

Based on these limitations, future studies should aim to study AA longitudinally and investigate the influence of disease severity, disease duration, disease status (inactive, remission, or relapse) and medication use on psychological wellbeing. These results would provide useful insights on potential at-risk groups in need of referral to psychological care.

The results of the current study highlight the impact of AA on psychological wellbeing. Clinicians treating people with AA should therefore be aware of the impact and refer to psychological care if needed. This could be accomplished through regular screening, for instance as part of value-based healthcare (120), or through the physician checking in on people’s mental health during outpatient clinic appointments.

## Conclusion

In summary, we have shown that living with AA has important consequences for psychological wellbeing. People with AA experienced worse psychological outcomes than healthy controls and comparable psychological outcomes compared to people with other dermatological diagnoses. Important challenges lay ahead on how to treat AA, both psychologically as well as medically.

## Data availability statement

The datasets analyzed as well as analysis scripts for this study can be found on the Open Science Framework (OSF): https://osf.io/fxt7p/.

## Author contributions

MD: conceptualization, methodology, formal analysis, writing—original draft, visualization, and project administration. KM: formal analysis and writing—review and editing. JK-O, JO, and SP: writing—review and editing. All authors contributed to the article and approved the submitted version.
