# Supplementary material for: Anxiety, depression, and quality of life in children and adults with alopecia areata: A systematic review and meta-analysis
Source: Front Med (Lausanne). 2022 Nov 29;9:1054898. doi: 10.3389/fmed.2022.1054898 (PMC9745337; doi:10.3389/fmed.2022.1054898)
Supplement: Supplementary file 1 [file Table_1.DOCX]

**Supplementary material 1 – Search terms per database**

**Embase**

('alopecia areata'/de OR (((alopecia OR area) NEXT/1 (areata OR circumscripta OR celsi OR universalis OR totalis))):ab,ti,kw) **AND** ('anxiety'/de OR 'social anxiety'/de OR 'anxiety disorder'/de OR 'depression'/de OR 'major depression'/de OR 'mood disorder'/de OR 'mental disease'/de OR 'emotional disorder'/exp OR 'quality of life'/exp OR 'emotional well-being'/de OR 'psychological well-being'/de OR 'social interaction'/de OR 'stigma'/de OR 'friendship'/de OR 'bullying'/de OR 'educational status'/de OR 'economic status'/de OR 'disease burden'/exp OR 'patient-reported outcome'/de OR 'quality of life assessment'/exp OR 'mental stress'/exp OR (HRQL OR HRQOL OR QOL OR PROM OR PROMs OR anxiet* OR depress* OR stress* OR burnout* OR burn-out* OR psychosocial* OR stigma OR friendship* OR bullying OR ((mood OR mental* OR psyc* OR emotion* OR externali* OR internali* OR affectiv*) NEAR/3 (disorder* OR diseas* OR dysfunct* OR handicap* OR symptom*)) OR ((qualit*) NEAR/3 (life*)) OR ((emotion* OR mood OR mental* OR psyc* OR social* OR educat* OR work* OR profession* OR school* OR economic* OR socioeconomic*) NEAR/3 (status OR well-being OR wellbeing OR function* OR experienc*)) OR ((social* OR romantic*) NEAR/3 (interaction* OR relation* OR function*)) OR ((disease* OR illness OR psychosocial*) NEAR/3 (burden*)) OR ((patient* OR self) NEAR/3 (report*)) OR ((shortform* OR short-form*) NEXT/1 (36 OR 12 OR 20 OR 8))):ab,ti,kw) NOT ((animal/exp OR animal*:de OR nonhuman/de) NOT ('human'/exp)) AND [english]/lim NOT ([Conference Abstract]/lim OR [preprint]/lim) NOT ('case report'/de OR 'case report*':ti)

**Medline**

(Alopecia Areata/ OR (((alopecia OR area) ADJ (areata OR circumscripta OR celsi OR universalis OR totalis))).ab,ti,kf.) AND (Anxiety/ OR Anxiety Disorders/ OR Depression/ OR Depressive Disorder, Major/ OR Depressive Disorder/ OR Mood Disorders/ OR Mental Disorders/ OR Quality of Life/ OR Social Interaction/ OR Social Stigma/ OR Friends/ OR exp Bullying/ OR Educational Status/ OR Economic Status/ OR Cost of Illness/ OR Patient Reported Outcome Measures/ OR exp Stress, Psychological/ OR (HRQL OR HRQOL OR QOL OR PROM OR PROMs OR anxiet* OR depress* OR stress* OR burnout* OR burn-out* OR psychosocial* OR stigma OR friendship* OR bullying OR ((mood OR mental* OR psyc* OR emotion* OR externali* OR internali* OR affectiv*) ADJ3 (disorder* OR diseas* OR dysfunct* OR handicap* OR symptom*)) OR ((qualit*) ADJ3 (life*)) OR ((emotion* OR mood OR mental* OR psyc* OR social* OR educat* OR work* OR profession* OR school* OR economic* OR socioeconomic*) ADJ3 (status OR well-being OR wellbeing OR function* OR experienc*)) OR ((social* OR romantic*) ADJ3 (interaction* OR relation* OR function*)) OR ((disease* OR illness OR psychosocial*) ADJ3 (burden*)) OR ((patient* OR self) ADJ3 (report*)) OR ((shortform* OR short-form*) ADJ (36 OR 12 OR 20 OR 8))).ab,ti,kf.) NOT (news OR congres* OR abstract* OR book* OR chapter* OR dissertation abstract*).pt. NOT ((exp animal/) NOT (human/)) NOT (Case Reports/ OR case report*.ti.)

**Cochrane**

((((alopecia OR area) NEXT/1 (areata OR circumscripta OR celsi OR universalis OR totalis))):ab,ti,kw) **AND** ((HRQL OR HRQOL OR QOL OR PROM OR PROMs OR anxiet* OR depress* OR stress* OR burnout* OR burn-out* OR psychosocial* OR stigma OR friendship* OR bullying OR ((mood OR mental* OR psyc* OR emotion* OR externali* OR internali* OR affectiv*) NEAR/3 (disorder* OR diseas* OR dysfunct* OR handicap* OR symptom*)) OR ((qualit*) NEAR/3 (life*)) OR ((emotion* OR mood OR mental* OR psyc* OR social* OR educat* OR work* OR profession* OR school* OR economic* OR socioeconomic*) NEAR/3 (status OR (well NEXT/1 being) OR wellbeing OR function* OR experienc*)) OR ((social* OR romantic*) NEAR/3 (interaction* OR relation* OR function*)) OR ((disease* OR illness OR psychosocial*) NEAR/3 (burden*)) OR ((patient* OR self) NEAR/3 (report*)) OR ((shortform* OR (short NEXT/1 form*)) NEXT/1 (36 OR 12 OR 20 OR 8))):ab,ti,kw)

**Web of Science**

TS=(((((alopecia OR area) NEAR/1 (areata OR circumscripta OR celsi OR universalis OR totalis)))) AND ((HRQL OR HRQOL OR QOL OR PROM OR PROMs OR anxiet* OR depress* OR stress* OR burnout* OR burn-out* OR psychosocial* OR stigma OR friendship* OR bullying OR ((mood OR mental* OR psyc* OR emotion* OR externali* OR internali* OR affectiv*) NEAR/2 (disorder* OR diseas* OR dysfunct* OR handicap* OR symptom*)) OR ((qualit*) NEAR/2 (life*)) OR ((emotion* OR mood OR mental* OR psyc* OR social* OR educat* OR work* OR profession* OR school* OR economic* OR socioeconomic*) NEAR/2 (status OR well-being OR wellbeing OR function* OR experienc*)) OR ((social* OR romantic*) NEAR/2 (interaction* OR relation* OR function*)) OR ((disease* OR illness OR psychosocial*) NEAR/2 (burden*)) OR ((patient* OR self) NEAR/2 (report*)) OR ((shortform* OR short-form*) NEAR/1 (36 OR 12 OR 20 OR 8)))) NOT ((animal* OR rat OR rats OR mouse OR mice OR murine OR dog OR dogs OR canine OR cat OR cats OR feline OR rabbit OR cow OR cows OR bovine OR rodent* OR sheep OR ovine OR pig OR swine OR porcine OR veterinar* OR chick* OR zebrafish* OR baboon* OR nonhuman* OR primate* OR cattle* OR goose OR geese OR duck OR macaque* OR avian* OR bird* OR fish*) NOT (human* OR patient* OR women OR woman OR men OR man))) AND DT=(Article OR Review OR Letter OR Early Access)

**Google Scholar**

“alopecia areata|circumscripta|celsi|universalis|totalis” “quality life”|”patient|self report|reported”|anxiety|depression|depressive
